# The relation between peptide hormones and sex hormone in patients with multiple sclerosis

**Published:** 2013

**Authors:** Elham Eftekhari, Masoud Etemadifar, Ali Ebrahimi, Shahrzad Baradaran

**Affiliations:** 1 Assistant Professor, Department of Physical Education and Sport Sciences, Islamic Azad University, Najafabad Branch, Isfahan, Iran; 2 Neurologist, Isfahan Eye Research Center (IERC) AND Department of Neurology, Medical School, Isfahan University of Medical Sciences, Isfahan, Iran; 3 Isfahan Research Committee of Multiple Sclerosis, Isfahan University of Medical Sciences, Isfahan, Iran; 4 Dr. Baradaran Medical Laboratory, Isfahan, Iran

**Keywords:** Ghrelin, Leptin, Testosterone, Multiple Sclerosis

## Abstract

**Background:**

Hormones can play a significant role in the pathogenesis of multiple sclerosis (MS). The aim of this study was to compare levels of ghrelin, leptin, and testosterone hormones of MS patients with healthy subjects, and assess the relationship between levels of peptide hormone and sex hormones in MS patients.

**Methods:**

35 MS patients with definite relapsing remitting multiple sclerosis (RRMS) (male = 9, female = 26) and 13 healthy subjects (male = 4, female = 9) were enrolled in the study. Levels of serum ghrelin, leptin, and testosterone hormones were measured in this study. ANOVA and Pearson correlation were used for data analysis (P < 0.05).

**Results:**

The female and male participants of the patient group were compared with the healthy group. No significant differences were found in serum of leptin, ghrelin, testosterone, ghrelin/leptin, and testosterone/leptin (P < 0.05). Spearman correlation coefficient showed that leptin had a significant negative correlation with the variability of testosterone (r = -1.00) in the healthy male group. Moreover, leptin had a significant positive correlation with the variability of BMI (r = 0.68) and weight (r = 0.59), at the 0.01 level (2-tailed), in the female patient group. In addition, in the healthy male group, ghrelin had a significant negative correlation with the variability of weight (r = -1.00).

**Conclusion:**

According to the results, there was no significant difference between peptide and sex hormones of MS patients and healthy persons. Furthermore, there was no significant relationship between peptide and sex hormones of MS patients and healthy persons.

## Introduction

Recently it has been shown that leptin, a cytokine-like hormone mainly secreted by adipocytes, can play a significant role in the pathogenesis of Multiple sclerosis (MS).^1, 2^

Leptin is a peptide hormone which regulates food intake, energy expenditure and metabolism.^3^ It also acts on the immune system cells by influencing the production of cytokines.^1, 2^ It has been previously observed that in RRMS (relapsing remitting multiple sclerosis) patients, leptin causes the production of cytokines by peripheral blood mononuclear cells (PBMC) in the acute phase of the disease and these changes in leptin serum levels are related to disease activity.^1, 4–6^

Ghrelin is functionally a natural leptin antagonist.^7, 8^ Recently, It has been shown that ghrelin as a polypeptide hormone exerts anti-inflammatory effects through inhibiting the secretion of both acute and chronic cytokines and chemokines in human endothelial cells, and mediates the opposite effects of leptin on peripheral immune responses.^9, 10^ In fact, ghrelin blocks the leptin-caused secretion of pro-inflammatory cytokines by human T cells.^10, 11^

The difference in serum leptin concentration of the two genders is well known.^12, 13^ Moreover, the results suggest that testosterone, as a gonadal hormone, may be one of the important regulators of leptin secretion.^14^ Recently it has been shown that testosterone therapy is safe and has potential neuroprotective effects in men with relapsing-remitting MS, and that muscle mass significantly increased after this treatment.^15^

Based on this evidence, the aim of this study was to compare levels of ghrelin, leptin, and testosterone hormones of MS patients with healthy subjects, and assess the relationship between levels of peptide hormone and sex hormone in MS patients.

## Materials and Methods

The protocol of this study was approved by the Ethics Committee of Al-Zahra Multiple Sclerosis Center, and all individuals gave written informed consents. 35 MS patients with definite RRMS and 13 Healthy subjects were enrolled into the study.

Exclusion criteria were as follows: presence of any concurrent psychiatric disease, regular drug use including oral contraceptives except for interferons or glatiramer acetate, presence of other diseases (neurological, endocrinological, rheumatological, hematological, acute or chronic infectious and/or inflammatory), liver or kidney dysfunction, or a body mass index (BMI) of over 28 Kg/m^2^. The patients and healthy groups matched for age, height, weight, and BMI. None of the healthy subjects had a history of autoimmune disorders, infection, or endocrine disease.

Levels of serum ghrelin, leptin, and testosterone hormones were measured in this study. Serum levels were measured in blood samples collected from each participant's antecubital vein at 8 a.m. after 12 hours of fasting. The blood samples were immediately centrifuged, and stored at -80°C until analyzed. The concentration of ghrelin hormone was measured through enzyme immunoassay using kits from EIA (Ghrelin “Human” EIA Kit, EK-031-31, 600808, Phonex Pharmaceuticals, Inc. California, U.S.A.). The concentration of leptin was measured through enzyme Immunoassay using kits from EASIA (KAP2281: 96 determinations BoiSource Europe S.A. rue the industry 8 B-1400 Nivelles Belgium). The concentration of total testosterone was measured through enzyme immunoassay using ELISA kits (DRG instruments GmbH, Germany division of DRG International Inc. Frauenbergstr.18, D-35039 Marburg.LOT:29K048-4). All samples from the tested subjects were analyzed using the IA counter (ELISA reader, stat fax). The Intra-assay coefficients of variation (CV) for blood samples were 2.2% for ghrelin, 3.6% for leptin, and 3.28% for testosterone.

### Statistical analysis

Data are expressed as mean ± SD. Statistical analysis was performed using ANOVA and followed by LSD post-hoc multiple comparisons test. The correlation between variables was calculated by Pearson correlation. P-values of less than 0.05 were considered to be statistically significant. Data were statistically evaluated with SPSS for Windows (version16; SPSS Inc., Chicago, IL., USA).

## Results

Demographic and clinical characteristics of all 48 participants of this study are given in Table 1. There were no significant differences at baseline in terms of weight, height, and BMI in males and females between the two groups (healthy and patients).

**Table 1:** Characteristics of the 13 male and 35 female subjects of this study are given as mean ± SD and independent sample t-test

Variables	Healthy male group mean ± SD (n = 4)	Patients’ male group mean ± SD (n = 9)	t	P	Healthy female group mean ± SD (n = 9)	Patients’ female group mean ± SD (n = 26)	t	P
Height (m)	1.77 ± 0.06	1.74 ± 0.04	1.02	0.33	1.63 ± 0.06	1.60 ± 0.05	1.8	0.07
Weight (kg)	74.50 ± 2.51	69.57 ± 5.44	1.68	0.12	60.77 ± 6.09	61.03 ± 8.97	0.08	0.93
BMI (Kg.m^2^)	23.60 ± 0.95	22.95 ± 2.24	0.53	0.60	22.72 ± 2.95	23.87 ± 3.53	0.87	0.37

P < 0.05, df = 11 for males, df = 33 for females

The female and male participants of the patient group were compared with the healthy group. There was no significant difference in serum of leptin, ghrelin, testosterone, ghrelin/leptin, testosterone/leptin (P < 0.05), which can be observed in Table 2.

**Table 2:** Measured parameters (mean ± SD) and Student's Independent t-test in measured variables in the male and female healthy subjects and patients with multiple sclerosis

Variables	Healthy male group mean ± SD (n = 4)	Patients’ male group mean ± SD (n = 9)	t	P	Healthy female group mean ± SD (n = 9)	Patients’ female group mean ± SD (n = 26)	t	P
Leptin (ng/ml)	5.07 ± 3.78	1.02 ± 1.23	-2.03	0.07	17.93 ± 1.41	16.45 ± 8.51	0.29	0.77
Ghrelin (ng/ml)	6.32 ± 2.40	8.53 ± 0.32	0.16	0.87	6.69 ± 1.51	7.15 ± 1.51	0.76	0.44
Testosterone (ng/ml)	6.88 ± 2.96	6.60 ± 2.07	1.7	0.10	0.39 ± 0.41	0.43 ± 0.13	0.26	0.79
Ghrelin/Leptin	1.97 ± 1.26	31.87 ± 37.7	1.58	0.21	0.60 ± 0.46	1.53 ± 3.31	0.83	0.42
Testosterone/Leptin	2.11 ± 1.41	30.53 ± 40.04	1.40	0.25	0.03 ± 0.03	0.08 ± 0.15	0.90	0.39

P < 0.05, df = 11 for males, df = 33 for females

To assess the relationships between leptin, ghrelin, or testosterone levels, and several variables, Spearman correlation coefficient analysis was carried out and adjusted for age, gender, BMI, leptin, ghrelin, and testosterone for four groups of healthy subjects and patients (Table 3).

**Table 3:** Relationships between hormone plasma levels as leptin, ghrelin, testosterone, and anthropometric parameters in healthy and multiple sclerosis subjects

Relationship	Healthy group M (n = 4)		Healthy group F (n = 9)		Patients’ group M (n = 9)		Patients’ group F (n = 26)	

	R	P	r	P	r	P	r	P
Leptin								
Ghrelin	0.31	0.68	-0.26	0.48	-0.17	0.70	-0.10	0.60
Testosterone	-1.00*		-0.05	0.89	-0.35	0.43	0.09	0.66
BMI	-0.40	0.60	0.35	0.35	0.64	0.11	0.68**	< 0.01
Weight	0.31	0.68	0.20	0.58	0.68	0.90	0.59**	0.01
Age	0.80	0.20	0.38	0.30	0.37	0.40	0.29	0.14
Ghrelin								
Testosterone	0.31	0.68	0.18	0.64	0.07	0.87	-0.35	0.07
BMI	0.31	0.68	-0.46	0.20	0.01	0.96	-0.15	0.44
Weight	-1.00*		-0.36	0.34	0.09	0.84	-0.09	0.64
Age	0.31	0.68	0.51	0.16	0.12	0.78	0.26	0.20
Testosterone								
BMI	0.40	0.60	-0.51	0.15	-0.03	0.93	0.44	0.02
Weight	0.31	0.68	-0.02	0.94	0.18	0.69	0.25	0.21
Age	0.80	0.20	-0.15	0.68	0.39	0.37	0.31	0.11

F = female, M = male, n= number

** Correlation is significant at the 0.01 level (2-tailed)

* Correlation is significant at the 0.05 level (2-tailed)

The Spearman correlation showed that, in the healthy male group, leptin had a significant negative correlation with the variability of testosterone (r = -1.00), and also in the patient female group, leptin was associated positively and significantly with the variability of BMI (r = 0.68) and weight (r = 0.59), at the 0.01 level (2-tailed).

In addition, in the healthy male group ghrelin was had a significant negative correlation with the variability of weight (r = -1.00).

## Discussion

In our study there was a significant difference in leptin levels. We found that leptin in females was higher than in males, and the mean plasma level of leptin in female patients was higher than in healthy female participants; this was not significant. Researchers concluded that males have lower plasma leptin concentrations than their female counterparts at any level of adiposity.^16–19^ Wauters has previously mentioned the existence of gender differences in leptin levels; leptin levels were found to be two to threefold higher in females than in males with the same BMI.^14, 17–19^ These differences reflect the difference in body composition between the two genders, and higher percentage of body fat and a higher ratio of subcutaneous to visceral fat in females.^14^ Furthermore, it was shown that females have higher cerebro-spinal leptin levels.^20^ Leptin acts through its receptor present on the immune cells, and supports the inflammatory response by causing the cascade of pro-inflammatory cytokines.^21, 22^ Therefore, two to threefold higher leptin levels in females than in males could be associated with the prevalence of MS in females.^34^ Munger et al. have shown that obesity at the age of 18 (BMI = 30 kg/m^2^) is associated with a greater than twofold increased risk of MS; weight during adolescence is one of the critical factors in determining risk of MS.^23^

The hypothesis is that obesity could be mediated by a low-grade chronic inflammatory state. Adipose tissue secretes cytokines that influence the immune system function, including leptin, and interleukin-6, both of which have been shown to reduce regulatory T cell activity.^24–27^ The elevation of leptin levels were inversely correlated with frequency of regulatory T cells in MS patients.^4^ There are several studies which suggest the pathogenesis of the effects of leptin hormone in MS patients.^5, 6, 22, 28^

Leptin may modulate the MS inflammatory process during relapses.^5^ In a study by Batocchi et al., an increase of leptin was observed before the first clinical exacerbation in relapsing patients, but after two months of INF-beta therapy, leptin significantly decreased.^6^ Therefore, they suggested that leptin be considered as a marker of disease activity.^6^ They found that leptin has serum levels in RRMS patients, in a stable phase of the disease, which is comparable with healthy subjects. However, there was no correlation between serum leptin and disease duration, EDSS (Expanded Disability Status Score), or number of relapses during the two years of the observation period.^6^

Another study reported that leptin caused interleukin-6, and interleukin-10 production by peripheral blood mononuclear cells (PBMCs) of patients in the acute phase of the disease; whereas, this has not been observed in patients in a stable phase or in healthy subjects. Moreover, they found no effect of leptin on monocytes in relapsing MS patients. Therefore, they concluded that leptin may modulate the MS inflammatory process during relapses.^1, 5^ Overall, the majority of the studies in this field emphasize the inflammatory effect of leptin on the immune system function.

We observed that gherlin levels in both male and female patient groups were lower than in healthy subjects, but this difference was not significant. Whereas, Berilgen et al. reported that the level of ghrelin in MS patients was significantly higher than in healthy subjects.^29^ Our findings did not confirm this. This difference may be related to the number of subjects. In the study by Berilgen et al. serum ghrelin levels were measured in 40 MS patients and 20 healthy subjects. They concluded that the increase in circulating ghrelin level may function against the proinflammatory process; thus, this increase may support the anti-inflammatory capacity of the patients. Another reason could be the difference in the subjects’ BMI (25 < BMI < 28kg/m^2^). They had eight subjects with BMI of higher than 25; 2 subjects were placed in the healthy group and 6 subjects in the patient group. The overall mean range of BMI in each group was less than 25 and no significant difference was observed between the two groups in terms of BMI at baseline. One of the most important points is the difference of fat distribution between the two genders, and the secretion of leptin from adipose tissue, and inverse relation between gherlin level and leptin level. In the study by Berilgen et al. there was no gender separation in patient and control groups; this could be a confounding factor in the statistical analysis results.^29^ Furthermore, one of the limitations of our study could be a smaller number of subjects.

In our study, no significant difference was observed between testosterone level of males and females in healthy and patient groups. In their study, Safarinejad concluded that the mean basal testosterone serum levels in MS patients were significantly lower than the mean for normal controls in male MS patients.^30^ Whereas, in the study by Tomassini et al. testosterone serum was significantly lower in the female MS group than the healthy subjects group.^31^ Moreover, strikingly, they stated that more brain lesions were detected by MRI in women suffering from MS who had the lowest testosterone concentrations.^31^ While men with relapsing-remitting or secondary progressive MS, and healthy men had generally similar sex hormone levels.^31^

The male MS patients had lower testosterone levels than healthy males.^32^ The hormone related modulation of pathological changes supports the hypothesis that sex hormones play a role in the inflammation and damage, and have a neuroprotective effect.^31^ Therefore, testosterone treatment could be beneficial for MS patients.^15, 33, 34^ The contradiction between these results and the results of our study could be due to the small sample size of our study. Testosterone plays an important role in the regulation of serum leptinlevels.^35^

Another dimension of our study was the relation between levels of peptide hormones, similar to toleptin and gherlin, and sex hormones such as testosterone. The correlations between sex hormone levels and the activity of cytokine-secreting immune cells from rodents as well as humans could lead to the idea that sex hormones directly influence the cytokine milieu in the immune system.^36, 37^ The results of this study indicated that there was a negative correlation between the level of ghrelin and leptin (r = -1.00) in healthy males, and also there was a positive correlation between leptin and BMI (r = 0.68), and leptin and weight (r = 0.59) in female patients. Moreover, there was a negative relation between ghrelin and weight (r = 1.00) in healthy males.

We found no correlation between leptin or gherlin and testosterone. However, Elbers et al. showed that the sex steroid hormones, in particular testosterone, are significant determinants of the difference in serum leptin levels of men and women.^35^ Leptin is not only produced primarily by adipocytes, but is also produced by T lymphocytes and neurons.^26, 38^ Several evidences indicate that leptin contributes to EAE (experimental autoimmune encephalomyelitis) /MS pathogenesis, influencing its onset and clinical severity.^4, 38^ Since the leptin plasma concentrations are proportional to the amount of fat tissue, obese/overweight individuals produce higher levels of leptin. Whether the increased MS prevalence in women vs. men, as mentioned earlier, is related to the worldwide increased prevalence of obesity and an enhanced immune sensitivity in women to leptin remains to be examined.^34^

The lack of a negative correlation between leptin and testosterone in patients, in our study, and the consistency of the results with other researches could be related to the small sample size of male subjects.

## Conclusion

Our data confirms the anti-inflammatoryrole of leptin, and its relation with the pathogenesis of MS. Increase in leptin serum levels activates the immune cells and causes the release of cytokines, which in turn are able to further increase leptin production and reverse the inflammatory process. Therefore, controlling the leptin level might slow down the ongoing progression of the disease.
